# The Protective Role Antioxidant of Vitamin C in the Prevention of oral Disease: A Scoping Review of Current Literature

**DOI:** 10.1055/s-0044-1786845

**Published:** 2024-07-16

**Authors:** Alessio Rosa, Alberto Maria Pujia, Claudio Arcuri

**Affiliations:** 1 Department of Chemical Science and Technologies, Dentistry, University of Tor Vergata, Rome, Italy; 2 Department of Biomedicine and Prevention, University of Rome Tor Vergata, Rome, Italy; 3 Department of Clinical Sciences and Translational Medicine, University of Rome Tor Vergata, Rome, Italy

**Keywords:** vitamin C, periodontal disease, nutrition, oral health, tooth

## Abstract

This review meticulously examined the connection between vitamin C and periodontal disease, as well as the potential of vitamin C to prevent this condition. To gather relevant data, comprehensive electronic searches were conducted across various databases, including PubMed, EMBASE, Cochrane Library, and Web of Science, focusing on studies that explored the relationship between vitamin C and periodontal disease in individuals aged between 18 and above. From an initial pool of 421 articles, 14 were ultimately chosen for detailed analysis. This selection encompassed seven cross-sectional studies, two case–control studies, two cohort studies, and three randomized controlled trials. The analysis of these studies revealed a consistent negative association between vitamin C intake, as well as its levels in the blood, and the incidence of periodontal disease across all seven cross-sectional studies. This indicates that higher vitamin C intake and blood levels are linked to a decreased risk of developing periodontal disease. In the two case–control studies, individuals suffering from periodontitis were found to have both a lower intake of vitamin C and reduced levels of vitamin C in their blood compared to those without the disease, further underscoring the potential protective role of vitamin C against periodontal disease. The progression of periodontal disease was observed to be more rapid in patients with lower dietary intake or blood levels of vitamin C compared to controls. Regarding the effects of vitamin C administration as an intervention, there was an improvement noted in gingival bleeding among patients with gingivitis; however, no significant benefits were observed in cases of periodontitis, specifically concerning alveolar bone absorption. Through the meticulous examination of available studies, this systematic review underscores the notion that adequate vitamin C intake and maintaining sufficient levels of vitamin C in the blood can contribute significantly to reducing the risk of periodontal disease.

## Introduction

Vitamin C (ascorbic acid) is a six-carbon lactone that is synthesized from glucose in the liver of most mammalian species, but not by humans, nonhuman primates, and guinea pigs, as they lack the enzyme gulonolactone oxidase. 1 So, human beings are bound to ingest vitamin C in their diet; if it did not occur, there would be a state of deficiency with a wide spectrum of clinical manifestations. Scurvy (clinical term for vitamin C deficiency) is a lethal condition if not properly treated. 2 Therefore, humans must ingest vitamin C to survive. Humans can obtain vitamin C only exogenously and mainly orally with subsequent gastrointestinal absorption and distribution. Vitamin C is mainly found in fruits and vegetables. L-ascorbic acid is an essential nutrient for all humans. The inability of the human body to produce this molecule is due to the absence of the enzyme L-gulono-1,4-lactone oxidase. 2 3 Vitamin C plays a crucial role in various metabolic processes within the human body, including the regulation of collagen, corticosteroids, neurotransmitter synthesis, iron absorption, and immune system responses. One of the key functions of vitamin C is facilitating the formation of collagen intermolecular cross-links, which strengthens the bonds between lysine and hydroxylysine in adjacent tropocollagen molecules. Without hydroxylation, the production of procollagen is reduced, leading to its probable accelerated degradation. Since vitamin C is essential for collagen stabilization, its deficiency can lead to collagen instability, affecting the periodontal ligaments and potentially leading to tooth loss. 4 5 6 Furthermore, L-ascorbic acid is crucial for endothelial cell function and promotes the proliferation of these cells, likely through its role in enhancing type IV collagen synthesis. The impact of vitamin C on various proinflammatory cytokines suggests its potential as a biomarker or adjunctive therapy. High doses of intravenous vitamin C have been shown to reduce proinflammatory cytokines and C-reactive protein in cancer patients, while low dietary intake of vitamin C is linked with increased inflammatory and oxidative stress. Although vitamin C has antioxidant properties, in high doses, it can act as an oxidant and selectively target cancer cells. Its effect on cell wall thickness via improved collagen fibers enhances vessel strength. 6 Vitamin C also promotes fibroblast migration in the skin and keratinocyte proliferation, potentially benefiting wound healing and reducing inflammation in the gums. Overall, previous research indicates vitamin C's role in reducing inflammation in periodontal tissues, preventing bacterial infections by enhancing neutrophil phagocytosis, and improving collagen synthesis and vessel strength, leading to better tissue regeneration. 7

An overwhelmingly large segment of the global populace, estimated at 90%, is afflicted by periodontal diseases, as highlighted by references. 7 8 When periodontitis advances, it devastates the alveolar bone that plays a crucial role in anchoring teeth, which can ultimately lead to tooth loss. Additionally, periodontitis is believed to elevate the risk for a range of other health complications, including type 2 diabetes mellitus and cardiovascular diseases, and it may negatively impact pregnancy outcomes. 8

Periodontal disease is characterized as an inflammatory condition that begins with a bacterial infection. This infection sets off an atypical response from the host's immune system, which is primarily responsible for the degradation of periodontal tissue. 4 5 A key player in the body's defense against periodontopathogens are the polymorphonuclear leukocytes (PMNs), which play a pivotal role in warding off these harmful pathogens. 5 6 These leukocytes initiate an antimicrobial reaction at the site of infection by activating several intracellular signaling pathways, including the production of reactive oxygen species (ROS). 7 While ROS are beneficial in controlling infections, their heightened levels can be detrimental, causing cytotoxic effects on the tissues supporting the teeth. 8 9 It is suggested that oxidative stress induced by PMNs could be a leading cause of tissue damage in periodontal diseases. 10 Evidence shows that individuals with periodontitis have higher levels of biomarkers indicating ROS-induced tissue damage compared to healthy controls. 11 12 13

Vitamin C, an essential nutrient known for its antioxidative properties, plays a significant role in neutralizing free radicals and acting as a cofactor for enzymes within cells. 14 15 Given its capacity to scavenge excessive ROS, vitamin C is considered a vital dietary antioxidant crucial for maintaining periodontal health. 16 Beyond its antioxidative function, vitamin C is instrumental in promoting the differentiation of progenitor cells in the periodontal ligament, which is vital for the prevention and management of periodontal disease. 17

**Fig. 1:**
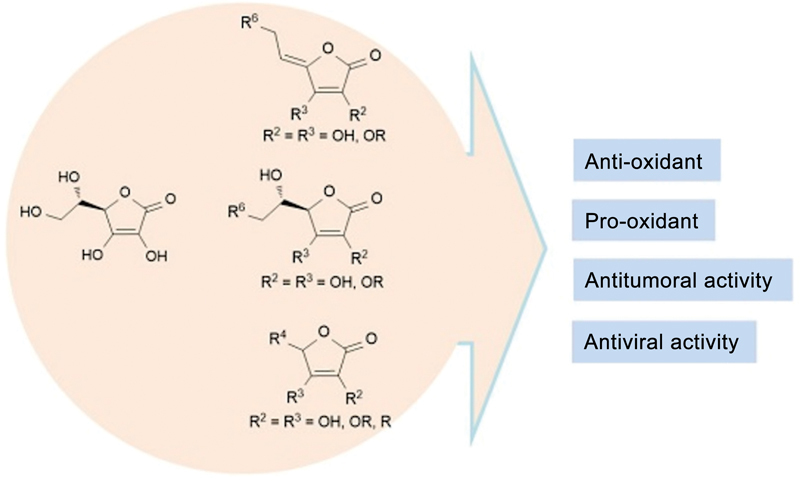
Antioxidant cellular activity of acid ascorbic. 5

In addressing the prevention of periodontal disease, it is crucial to produce evidence on nutritionally effective strategies. Over the past two decades, a wealth of epidemiological research has been undertaken to explore the link between vitamin C consumption and periodontal disease. These studies have also delved into vitamin C's potential in preventing periodontal disease, with measurements typically based on dietary intake or blood levels of vitamin C. It is important to note that periodontal disease encompasses both gingivitis and periodontitis, each with distinct pathological features and possibly varying relationships with vitamin C intake. Despite the abundance of studies, a comprehensive systematic review focusing on the association between vitamin C's dietary intake and blood concentration with periodontal disease, as well as its preventive capabilities, has yet to be conducted. This study, therefore, seeks to fill this gap by systematically reviewing the available literature on the subject ( Fig. 1 ).

## Methods

For this comprehensive review, the criteria for inclusion were meticulously established in alignment with the Preferred Reporting Items for Systematic Reviews and Meta-Analyses (PRISMA) guidelines, adopting the structured approach of the population (P; specifically targeting “human adults”), intervention or exposure (I; focusing on the “impact of vitamin C on periodontal disease”), comparison (C; comparing “various levels of vitamin C intake, different concentrations of vitamin C in the blood, or the absence of vitamin C supplementation”), and outcome (O; evaluating “parameters indicative of periodontal disease”) model, widely referred to as the PICO model. 18 To ensure a thorough and unbiased assessment of the studies, two independent researchers undertook the task of evaluating the eligibility of each study. This preliminary screening process involved a careful review of both the titles and abstracts of potential studies, guided by the PICO model's framework. The central question guiding this process was: “Is there an association between vitamin C and periodontal health status?.”

Additionally, to be considered for inclusion in this systematic review, studies were required to meet several specific criteria: (1) the study must be authored in English, (2) the publication date falls within the timeframe from July 1998 to June 2018, (3) the focus of the study is on exploring the association between periodontal disease and vitamin C, (4) the study subjects are adults, with an age requirement of 18 years or older, and (5) the study employs quantitative methodologies for the collection of data. This inclusion criteria set aimed to encompass epidemiological studies involving adult populations. The exclusion criteria were the coexistence of other pathologies in samples of adult patients, animal studies, *in vitro* studies, non-English studies, systemic reviews with meta-analysis, letters, dissertations, and abstracts.

To gather the necessary literature for this review, an extensive search was conducted across several key databases: PubMed, EMBASE, Cochrane Library, and Web of Science. The search strategy employed specific terms aimed at capturing relevant studies, using combinations such as “periodontal disease and vitamin C” or “periodontal disease and ascorbic acid” to ensure a comprehensive collection of pertinent research on the subject ( Table 1 ).

**Table 1:** Search strategy with Mesh term

***PubMed*** Scurvy AND periodontal disease (“periodontal disease”[MeSH Terms] OR (“vitamin C”[All Fields] AND “periodontal disease”[All Fields]) OR “scurvy”[All Fields]) AND (“vitamin C”[All Fields] OR “scurvy”[MeSH Terms] OR (“periodontal disease”[All Fields] AND “dentistry”[All Fields]) OR ” dentistry”[All Fields])
***Web of Science*** (vitamin C) AND (periodontal disease) (ALL FIELDS) ***Scopus***
(vitamin C) AND (periodontal disease)TITLE ABS KEY

### Selection Criteria and Data Extraction

**Fig. 2:**
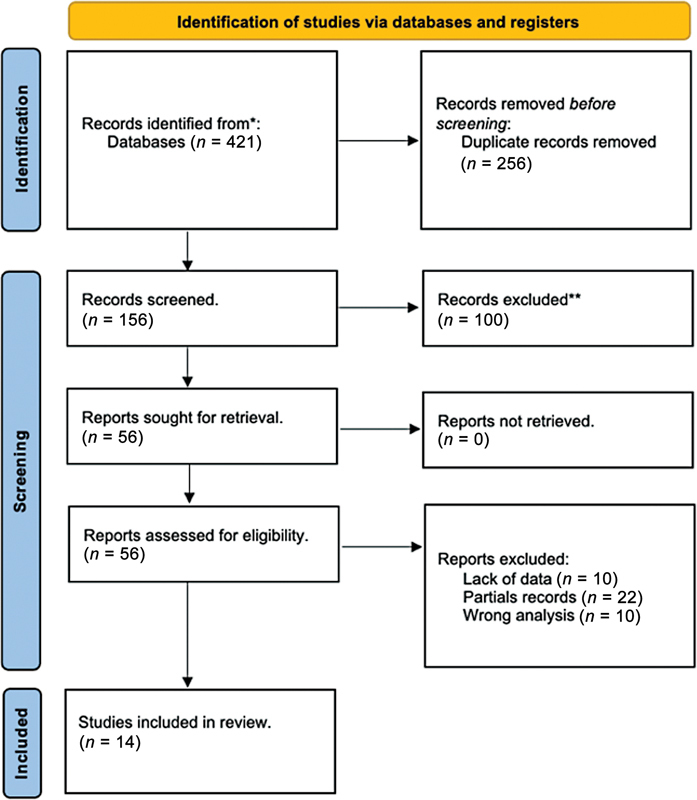
Search strategy flowchart.

Fig. 2 shows the entire selection and search strategy for articles. The research produced 421 articles. Two authors first independently evaluated the studies identified for compliance with the inclusion and exclusion criteria. Next, the full texts of these articles were examined. Most of these studies were excluded because they did not analyze correlations between vitamin C and periodontal disease. After removal of duplicate records and records that did not meet the inclusion criteria, 14 publications were selected.

## Results

The initial search across various databases for relevant literature on the topic resulted in the identification of 421 studies. A meticulous manual search process was implemented to retrieve these records. Subsequent to the elimination of duplicates, a total of 156 records were considered for screening. Following a preliminary review based on titles and abstracts, 110 of these records were deemed unrelated to the scope of the review and were thus excluded. This left 46 articles, which underwent a more detailed analysis to assess their relevance further.

Of these 46 articles deemed potentially relevant, 32 were found not to meet the specific inclusion criteria established for this review. Consequently, 14 studies were ultimately selected to be part of this systematic review, as delineated in the presented flowchart. The composition of these selected studies includes seven cross-sectional studies, 19 20 21 22 23 24 25 two case-control studies, 26 27 two cohort studies, 28 29 and four randomized controlled trials (RCTs). 18 30 31 Among the studies not classified as RCTs, six investigated vitamin C intake through dietary analysis, while another six studies focused on measuring the concentration of vitamin C in the blood. Interestingly, there was one study that provided insights into vitamin C levels from both dietary intake and blood concentration perspectives. The RCTs specifically examined changes in periodontal tissue conditions by comparing metrics before and after the administration of vitamin C.

The criteria used to assess periodontal disease in these studies were comprehensive, including various indicators such as the community periodontal index (CPI), pocket depth (PD), attachment loss (AL), bone loss, clinical attachment level (CAL), bleeding on probing, gingival index (GI), gingivitis severity index, and sulcus bleeding index (SBI). These indicators collectively provided a holistic view of the periodontal health status across the studies included in this review.

Several studies have delved into the connection between the consumption of vitamin C and the prevalence of periodontal disease, yielding insightful findings. In the investigation conducted by Lee at al, it was discovered that adults aged 19 and above who consumed the most vitamin C, specifically more than 132.2 mg/day (falling into the highest quartile of intake), exhibited significantly lower scores on the CPI in comparison to those whose intake was at or below 47.34 mg/day, placing them in the lowest quartile of consumption. Similarly, the research by Park et al highlighted 6 that within a demographic of women and nonsmokers aged between 19 and 39 years, individuals with a vitamin C intake below the median value of 81.3 mg/day were found to have a substantially higher proportion of CPI scores equal to or greater than 3, in contrast to their counterparts who consumed more vitamin C.

Additional large-scale investigations have underscored significant correlations between the dietary intake of vitamin C and indicators of periodontal health. Tanaka et al reported that a lack of sufficient vitamin C intake was linked to more severe manifestations of periodontal disease. 20 Concurrently, another study established that a decreased intake of dietary vitamin C was associated with an elevated risk of developing periodontal disease. 22 Focusing on the relationship between CAL and the levels of vitamin C in serum/plasma, three studies have provided valuable insights. Specifically, two of these studies demonstrated a negative correlation between the extent of AL and plasma vitamin C levels, indicating that lower levels of plasma vitamin C were associated with greater AL among participants. Bartold and Van Dyke found that serum vitamin C concentrations were inversely related to the degree of CAL, 24 suggesting that higher serum vitamin C levels may protect against AL. Additionally, Nazir reported that the occurrence of severe periodontitis was significantly more common in individuals with serum vitamin C levels below 8.52 mmol/L compared to those with higher levels of vitamin C. 23 Moreover, Gokhale et al confirmed a negative correlation between the degree of AL and plasma vitamin C levels among the examined subjects, 17 further affirming the protective role of vitamin C against periodontal deterioration.

In two distinct case–control studies, identified as 26 27 the levels of serum vitamin C were scrutinized by comparing individuals diagnosed with periodontal disease against healthy control participants. The findings from both studies unanimously indicated that those suffering from periodontitis exhibited notably lower levels of serum vitamin C in comparison to the healthy control group.

Furthermore, the efficacy of vitamin C supplementation as a preventive measure against periodontal disease was explored through four specific studies. Among these, one study delved into the impact of locally applied vitamin C, through the use of a dentifrice infused with ascorbic acid, on the health of periodontal tissues. 20 This particular study observed that the application of the ascorbic acid-containing dentifrice led to a marked improvement in reducing symptoms of gingivitis. 22 Another investigation focused on the consumption of grapefruit and its effect on periodontal health, revealing that individuals with chronic periodontitis experienced a significant improvement in their sulcus bleeding index following grapefruit intake. 26

Additionally, two other studies employing clinical trial methodologies assessed the combined effects of vitamin C supplementation and nonsurgical treatments for periodontal disease. In the study conducted by Gokhale et al, it was demonstrated that the conjunction of nonsurgical periodontal treatment with vitamin C supplementation resulted in a significant decrease in the SBI among patients diagnosed with gingivitis. 29 However, it was noted across these studies that vitamin C supplementation did not confer any additional benefits toward the improvement of clinical measures in cases of periodontitis, 28 29 suggesting a nuanced role of vitamin C in periodontal disease management, particularly emphasizing its beneficial effects in the early stages of gingival inflammation rather than in advanced periodontitis.

## Discussion

All the cross-sectional studies included in this review uniformly found a significant link between periodontal disease and either the dietary intake of vitamin C or its blood levels. Specifically, in two case–control studies 26 27 individuals diagnosed with periodontitis had noticeably lower serum vitamin C levels compared to their healthy counterparts. These observations suggest a mechanism wherein vitamin C, derived from dietary sources, is transported to the periodontal tissue through the bloodstream, thereby diminishing the risk of periodontal disease development. Nonetheless, an alternative interpretation arising from the cross-sectional studies proposes a reverse causality where the observed associations might reflect the impact of periodontal disease on vitamin C intake, possibly due to compromised mastication abilities, rather than the influence of vitamin C intake on periodontal health. The need for longitudinal studies is emphasized to clarify and confirm these associations. 23

Within this review, two cohort studies 28 29 provided evidence supporting a longitudinal relationship between vitamin C intake and periodontal disease, reinforcing the hypothesis that dietary vitamin C mitigates inflammatory responses in periodontal disease. However, the reliance on the same population sample in these two cohort studies calls for further research through additional cohort studies to solidify the reliability of this observed relationship.

Among the three RCTs discussed, two 13 14 indicated positive outcomes on periodontal health indices such as the GI, SBI, and PD, attributed to vitamin C supplementation. These improvements, particularly in gingival conditions, underscore vitamin C's potent antioxidative properties, which are thought to reduce oxidative stress in gingivitis. Moreover, vitamin C's capability to lessen the cytotoxic and apoptotic activities of *Porphyromonas gingivalis* in periodontal ligament cells and gingival fibroblasts may contribute to these benefits. However, these positive effects were not observed in patients with periodontitis, suggesting that vitamin C's antioxidative potential might be inhibited by factors produced when inflammation spreads to deeper periodontal tissues, including the alveolar bone.

Despite these findings, vitamin C supplementation did not show effectiveness in improving PD, echoing the results of another study that also reported no benefit in PD and attachment level improvement. This suggests that while vitamin C can induce osteogenic differentiation of periodontal ligament progenitor cells *in vitro* , its ability to stimulate alveolar bone regeneration *in vivo* remains unreported, likely explaining the observed lack of impact on PD reduction following vitamin C supplementation. 31

The intervention studies reviewed do present evidence suggesting that vitamin C supplementation may offer some benefits in managing periodontal disease, supporting the nutrient's preventive potential against this condition. Furthermore, the consideration that periodontopathic pathogens might decrease blood vitamin C levels through biodegradation is challenged by a study indicating that serum ascorbic acid levels in patients with moderate-to-severe periodontitis remain unchanged after scaling and root planning treatment, suggesting that while vitamin C levels can influence periodontal health, the reverse is not true. 25

The review also touched on the effects of other antioxidative vitamins, like vitamin A and E, on periodontal disease, yet only vitamin C consistently showed an association with periodontal health improvement, underlining the significance of focusing on vitamin C's relationship with periodontal disease. 30

Complex interactions between vitamin C, diabetes, and periodontal disease are highlighted, with studies suggesting that diabetic conditions might affect the efficacy of vitamin C due to glucose's interference with vitamin C transportation to cells and stimulation of the hexose monophosphate shunt. Despite these potential interactions, evidence indicates that dietary or supplemented vitamin C remains effective, especially in improving the immune function and potentially benefiting diabetic patients' periodontal status. 23

The review also delves into the varying impacts of vitamin C on periodontitis among smokers and nonsmokers, suggesting complicated interactions between vitamin C's antioxidative effect and oxidative stress induced by tobacco on periodontal tissues. The need for further research to unravel these interactions is emphasized. 24

Lastly, the review discusses limitations such as the diversity in periodontal disease indicators used across studies, which hinders direct comparisons of study results. It also notes the influence of confounding factors like smoking and diabetes on the relationship between vitamin C and periodontal disease, acknowledging the current evidence as perplexing and calling for more extensive research to clarify these associations. 21

## Conclusion

This review has significantly enhanced the body of scientific knowledge by providing a comprehensive analysis of the relationship between vitamin C consumption and the occurrence of periodontal disease. It underscored the preventive role of vitamin C in halting the advancement of periodontal disease. Nevertheless, there is a pressing need for more detailed investigations concerning the application of periodontal indicators and a thorough delineation of the elements that affect this relationship, to deepen the understanding of the connection between vitamin C and periodontal disease.

Future research directions include the necessity to standardize the indicators used to measure periodontal disease, enabling more precise comparisons across different studies. Additionally, it is imperative to delve into how smoking and diabetes might impact the effectiveness of vitamin C in preventing periodontal disease. The review also highlights the potential benefits of fostering collaborations between oral health practitioners and dietitians to boost oral health promotion efforts within communities. Moreover, it advocates for research into the synergistic effects of vitamin C and chlorhexidine on the prevention and treatment of periodontal disease. This thorough examination not only broadens the scientific discourse on the preventive capacity of vitamin C against periodontal disease progression but also calls for further inquiry into the utilization of periodontal indicators and the clarification of factors influencing this correlation, to augment the research on the correlation between vitamin C intake and periodontal disease.
